# Long-Term Evaluation of Bone Healing Monitoring Using an Instrumented Plate with Measurement Sensors (Smart Implant) over 10 Years

**DOI:** 10.3390/s25185779

**Published:** 2025-09-16

**Authors:** Arndt P. Schulz, Birgitt Kowald, Matthias Münch, Klaus Seide, Nils Weinrich, Tobias Barth, Benjamin Kienast

**Affiliations:** 1 Center for Clinical Research, BG Klinikum Hamburg, Bergedorfer Str. 10, 21033 Hamburg, Germanym.muench@bgk-hamburg.de (M.M.); n.weinrich@bgk-hamburg.de (N.W.); t.barth@bgk-hamburg.de (T.B.); 2 Section Medicine, Universität zu Lübeck, Ratzeburger Allee 160, 23538 Lübeck, Germany; 3 Fraunhofer IMTE, Mönkhofer Weg 239a, 23562 Lübeck, Germany; 4 Department of Trauma & Orthopedics, BG Klinikum Hamburg, Bergedorfer Str. 10, 21033 Hamburg, Germany

**Keywords:** bone fracture healing, smart implant, force sensor, fracture monitoring, instrumented implant, nonunion, telemetry, locked bone plate

## Abstract

**Highlights:**

**What are the main findings?**

- With first- and second-generation telemetric bone implants, force transmission over the fracture region is possible.
- By this, the status of bone union over time can be monitored.

**What is the implication of the main finding?**

- Healing process of bone union can successfully be monitored by smart implants.
- Long-term monitoring is possible when using external power sources.

**Abstract:**

A total of 66 smart implants were included. As a measure of bony stability, the relative elastic compliance of the osteosynthesis was determined from the gradient between the applied external load and the measured implant load over the entire healing process. The healing process of non-unions of the femur with a smart implant was tracked by telemetric measurements over a timespan of up to 10 years. The measurements of the longest healing process show a very slow but constant decrease in force transmission over the implant, radiological findings over 10 years show corresponding consolidation until bony healing. The use of a telemetrically instrumented bone plate, a so-called smart implant, to monitor the healing process is a successful procedure to support the clinician in his decision to take further surgical measures or to wait until healing occurs.

## 1. Introduction

Implants for bone fracture healing monitoring could alter fracture treatment in the future as they do not rely on subjective radiological and clinical examination. In our institution, instrumented plate with measurement sensors (smart implants) are under assessment since about 20 years prior to gain information regarding the bone healing process. We report here the long-term functionality of such devices.

Patients with non-union of the femur show a very low health-related quality of life with a time-trade-off score of 0.68 [1]. This ranks significantly behind diseases such as type I diabetes (0.88) or stroke (0.81) [2].

In addition to the psychological burden for the patient due to the long duration of the healing process, combined with pain and repeated hospitalization [3], non-union also poses a problem in terms of socioeconomic aspects [4,5]. Non-unions cause high treatment costs [6,7,8,9], which is between 2.6 and 4.3 times that of an uncomplicated fracture [10], and are among the complications in the treatment of fractures [11].

There is currently no standardized definition of non-union. The 6-month limit is widely used, after which non-union is generally considered to be present. The American FDA (U.S. Food and Drug Administration) only assumes non-union after 9 months [12]. To date, the classification according to Weber and Cech from 1976 is most commonly used to assess non-union [13]. Here, the non-union is assessed in the radiographic image in two planes based on the extent of callus formation. Typically, the healing process is documented and controlled by radiographs, although it is known that only an uncertain assessment of the mechanical situation of the healing bone can be made radiographically [14,15].

As a result, intensive research is being carried out into additional options for optimizing the assessment of fracture healing, such as indirect radio-stereometric analyses, vibration analyses, ultrasound examination techniques and even the evaluation of patients’ movement and loading patterns [16,17]. A system for telemetric fracture healing monitoring is currently in the clinical validation process for CE-approval (Clinical Trials.gov Number NCT05410587). In this study, technical functionality as well as the relationship between relative implant load and bone healing status will be assessed over a period of the initial 6 months.

A telemetric system was developed at the BG Klinikum Hamburg that uses an instrumented internal fixator—smart implant—to indirectly measure the healing process quantitatively and mechanically [18,19,20,21] using strain sensors. This system was used in patients with previously therapy-resistant non-unions of the femur in the timeframe of 2005–2016, so that quantitative clinical data are now available for a biomechanical analysis of bone healing. A total of 66 telemetrically instrumented locking plate fixators (or smart implants) were used in 61 patients.

Out of 66 cases, there were 4 cases with unusually long healing times. These cases are particularly interesting because consolidation only took place after a very long time. A particular advantage was that measurements with the instrumented implant could be used to decide on the best treatment form [22].

Major objective of this study was to investigate the long-term function of such embedded systems in bone osteosynthesis. This includes a description of the performance as well as the complications occurring during treatment.

## 2. Materials and Methods

### 2.1. Study Subjects

The total patient population consisted of 50 men (82%) and 11 women (18%). The average age of the men was 40 years (SD 13.15) and that of the women also 40 years (SD 20.46). All patients had developed non-union and were operated 8–23 months after the initial accident.

A total of 66 telemetrically instrumented locking plate fixators—smart implants—were used in 61 patients with non-unions. In five cases, the implant had to be exchanged due to malfunction or osteosynthesis failure. In 40 implants, measurement was possible for the full time of fracture healing. Some of the cases had been presented in previous publications [22,23,24].

### 2.2. Material

**Figure 1:**
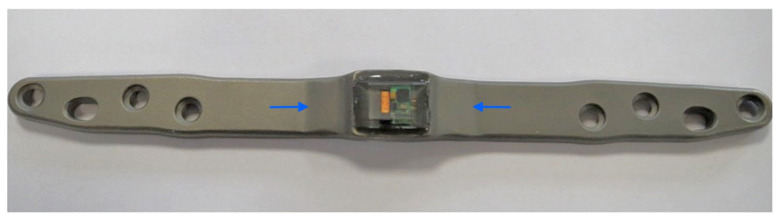
Instrumented locked plate with telemetry electronics attached. Blue arrows mark the thickened area.

**Figure 2:**
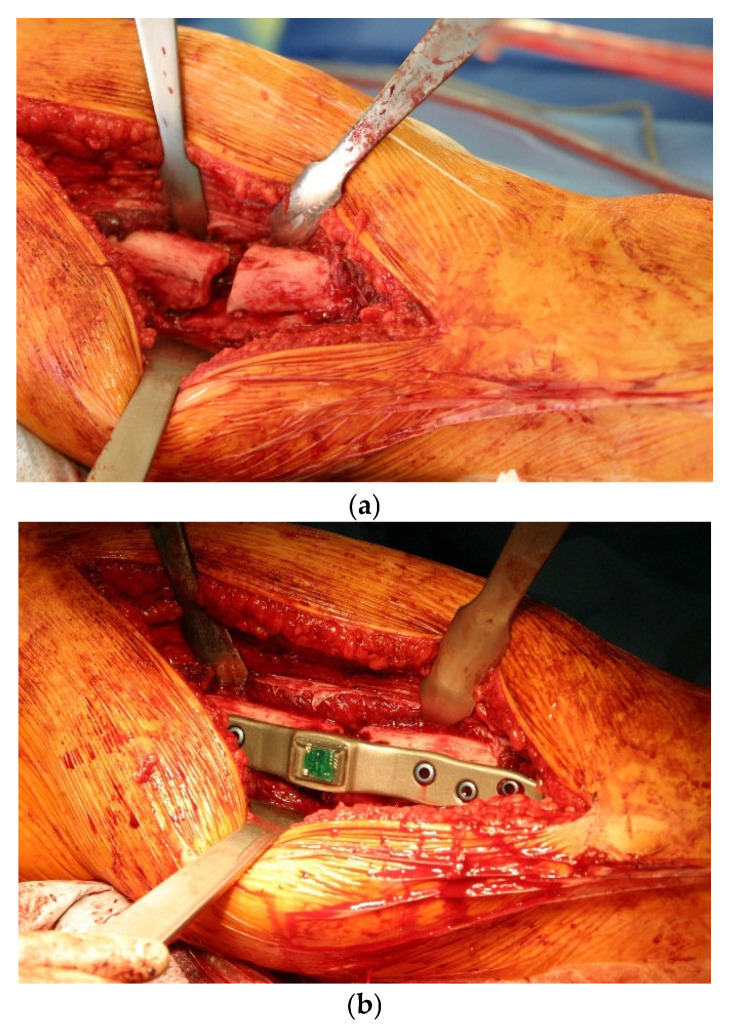
Intraoperative images before (**a**) and after osteosynthesis (**b**) with the smart implant.

The instrumented implant was based on a commercially available osteosynthesis plate (litos/GmbH, Ahrensburg, Germany), which was modified so that a thickening was constructed in the shaft area to which the telemetry electronics were attached (Figure 1) to allow safe attachment of strain sensors. Figure 2 shows the intraoperative image before (a) and after (b) the treatment with the smart implant. Osteosynthesis was conducted using a femoral locked plate that measures 200 mm in length, 20 mm in width, and 6.5 mm in thickness, featuring a waveform over the fracture site.

The telemetric system included a telemetry module attached to an internal locking plate, an external reader with an antenna, a force sensor, and a laptop for processing. The module featured a strain gauge bridge on the plate’s rear, coated in epoxy resin, and a 10 mm × 12 mm circuit board with a microprocessor. It was powered electromagnetically by a battery-free reader unit (10 cm antenna, 8 × 5 × 2 cm electronics) placed on the skin. The force sensor was a strain gauge model N2A-06-S1449-1KB/E2 (ME-Meßsysteme GmbH, Hennigsdorf, Germany) is a high-precision sensor designed for structural and materials testing applications. The strain gauge has a grid length of 1.78 mm and a nominal resistance of 1200 Ohms with a tolerance of 15%. Its connection is facilitated through soldering pads for reliable electrical and mechanical contact. The sensor’s sensitive element is made of metal foil, offering excellent stability and durability over a wide range of operating conditions. The carrier material is polyimide, which provides a flexible and robust base suitable for various surfaces. Temperature compensation is specifically adapted for metal structures, following the standard steel-06 compensation curve, making this strain gauge particularly suitable for implant monitoring.

An external force sensor was linked to the computer for data storage and analysis, facilitating real-time evaluation of implant load versus applied load to assess bone healing. Varus and valgus stress were measured at the knee with a hypomochlion in a similar manner.

**Figure 3:**
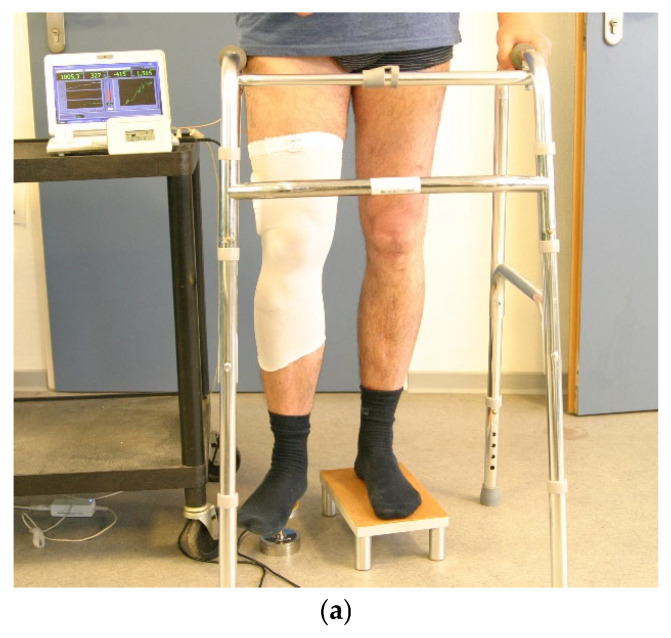

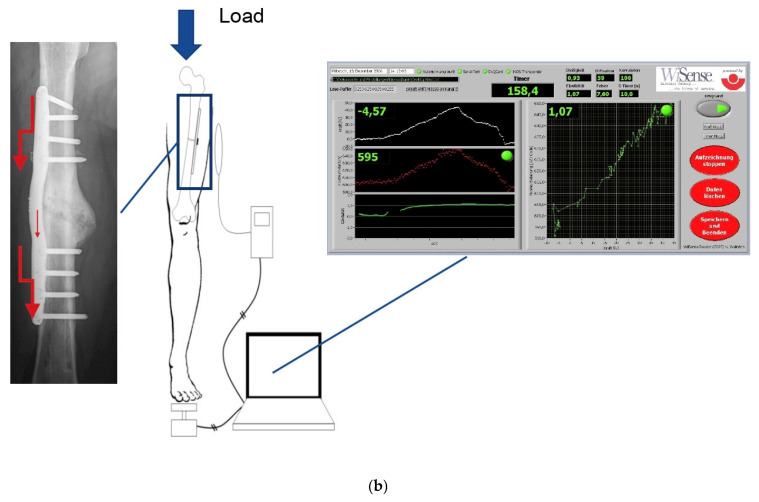
(**a**) Measurement under axial load in standing position. Force sensor under the right foot. (**b**) Diagram of the setup in (**a**).

The measured value was recorded transcutaneously using a reader (see Figure 3 standing in front of the laptop). A force sensor positioned under the patient’s heel was used to measure the external load on the limb, with the measured values being recorded in the laptop computer in parallel with the measurement signals transmitted by the instrumented implant (Figure 3).

Specially developed software enabled the continuous recording and display of the measured implant value as well as the simultaneous measurement of the load applied to the extremity. This led to the direct display of the correlation between the externally applied load and the measured implant value with the display of the regression line.

### 2.3. Methods

#### Definition of Healing Status

The measurements were taken in the laboratory for biomechanics at the BG Klinikum Hamburg at intervals of 2–4 weeks for 6 months, then at intervals of 3 months for 4 years; after this only one patient remained, and the intervals were set annually. Intervals were lengthened after 6 months as there was only a minor difference in 4 weeks of measurements at the end of the first 6 months. Four different measurement setups were performed. Next to axial load while standing (Figure 3), and varus, valgus and axial load while lying was measured. All measurements were evaluated separately.

As a measure of stability, the relative elastic compliance of the osteosynthesis was determined from the gradient between the applied external load and the measured implant load [19] using strain sensors. It was calculated using the Excel function “Trendline/linear” and describes the elasticity of the overall system consisting of plate, bone and callus (note: the reciprocal value of the elasticity corresponds to the stiffness of the system). With increasing consolidation, the load transfer from the plate to the bone shifts so that a lower load is measured in the plate for the same external load and the gradient becomes smaller.

Radiographs and computerized tomography (CT) scans were also taken as specified by the surgeon treating the patient in the outpatient clinic. Radiological healing was defined as union of at least 3 cortices.

## 3. Results

In 61 patients, the implant was successfully implanted. The procedure was performed in 50 men (82%) and 11 women (18%).

The average age of the men was 40 years (SD 13.15) and that of the women also 40 years (SD 20.46). All patients had developed non-union and were operated 8–23 months after the initial accident (mean 12.4 months).

In all cases, initial measurements were possible in the first 6 months. There were 12% surgical complications and 19.5% technical problems registered during this study. Planned removal of metalwork was not seen as complication; this was performed in 60 patients. The material was removed after a mean of 3 years (SD 1.55) in 60 patients after fracture healing occurred (52 cases) or a surgical complication made a revision necessary. The median time to clinical healing was 14 weeks (first quartile 9.8 weeks, third quartile 21.25 weeks).

In 13 cases, the healing process could only be partially measured due to technical problems with an initially non-optimal measurement range as well as mechanical problem with the titanium–epoxy-interface of the electronics. This only applied to the first-generation implants in the initial phase (2005–2010, *n* = 33). After that, in the second generation, the measuring range was extended. For the first generation, 1024 digits measuring range (2^10^) were available; for second generation this was increased to 32,768 digits (2^15^). In the event of plastic deformation, it could happen with first-generation implants, that the zero point was displaced too far, causing it to hit the upper or lower range.

Regarding the interface, the connection between epoxy and titanium was a mechanical problem. This was then addressed by surface treatment (blasting with corundum) before mounting of the electronics. In addition, a base for the electronics was introduced to prevent plastic bending in the electronics area.

In eight cases, a revision procedure, other than the planned removal of metalwork, was performed (13.1%). In three cases of these a plate fracture occurred, in one case a plate bending, two times a screw loosening, once intraoperatively a surgical torsion error of the femur occurred that was only seen after the operation and once a change to a prosthesis due to a periprosthetic fracture between the smart implant such that a knee prosthesis was required. One patient healed after 10.5 years. Metal removal has not yet taken place in this patient.

**Figure 4:**
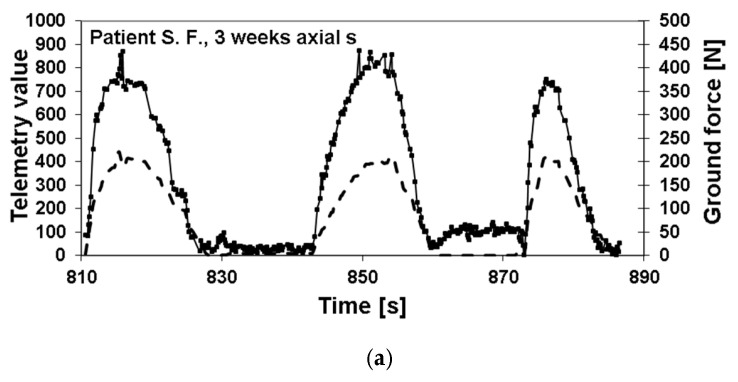

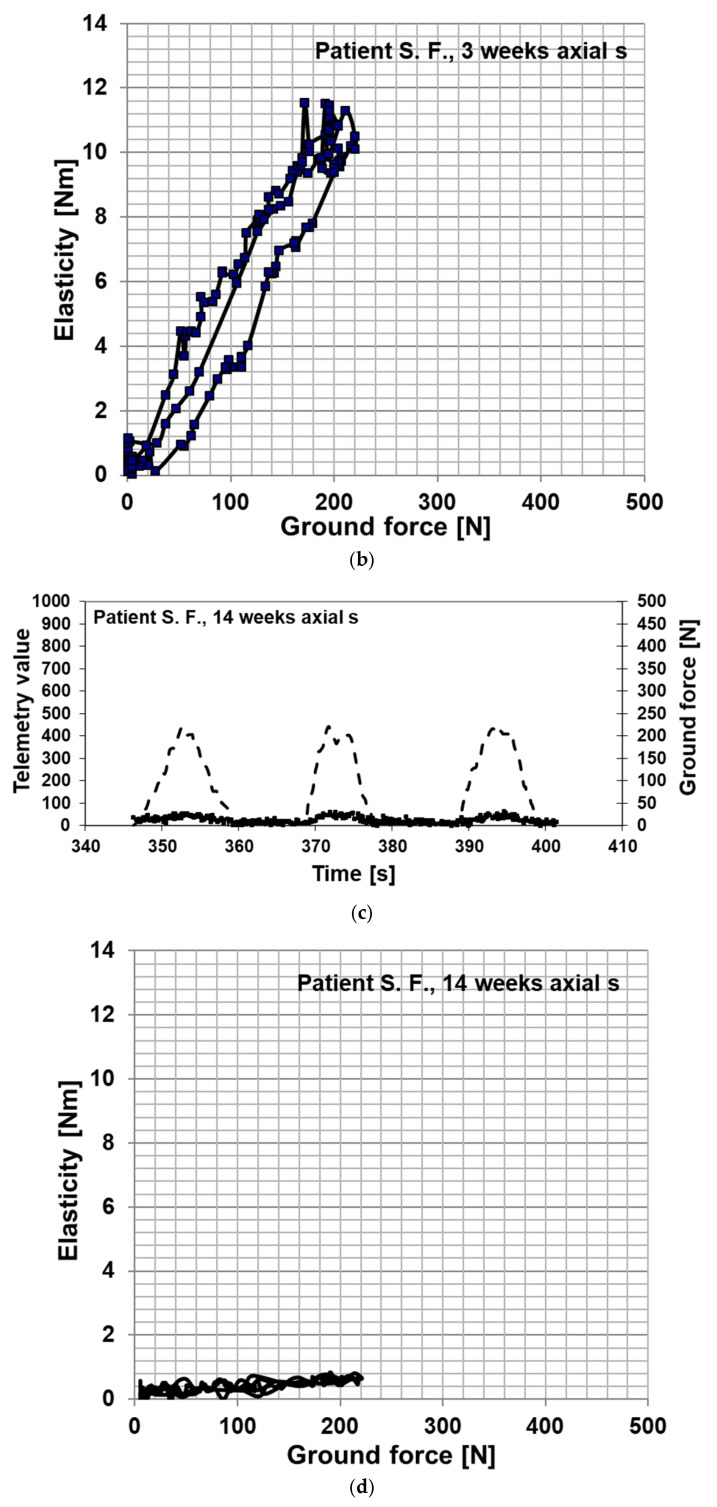
Example of original recordings of externally applied load (dotted line) and telemetrically measurement implant load (solid line). Three weeks postoperatively (**a**), high implant loads were measured; the implant load was nearly gone by 14 weeks (**c**). Each measurement consisted of three cycles of increasing and decreasing external load. The corresponding slope of the regression line between externally applied load and measured fixator loads (**b**,**d**) was calculated as the parameter of bone healing.

In Figure 4 is an example depicted of the healing process of a patient with a healing time of 14 weeks, which is about the average time to healing in this study (Figure 4a,c) including calculation (Figure 4b,d).

**Figure 5:**
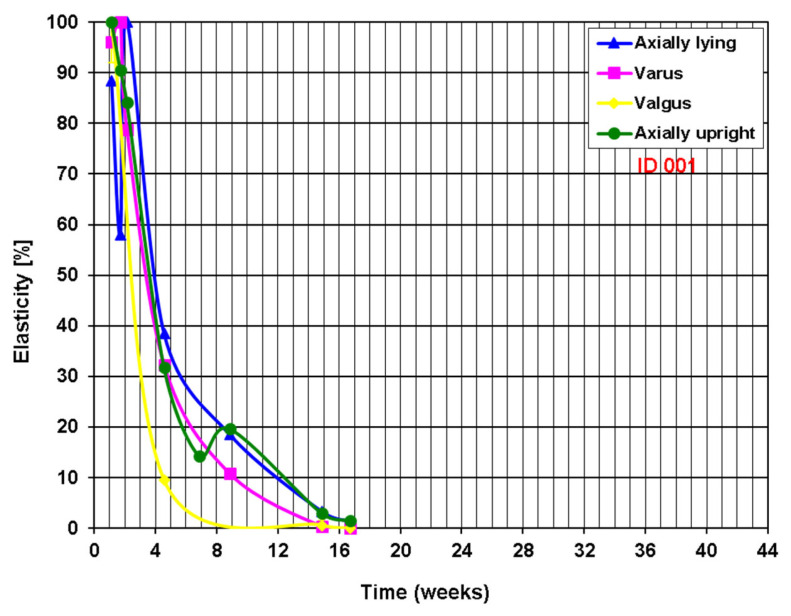
Curve of the telemetrically measured elasticity of the osteosynthesis. The different measuring modalities (lying, varus, valgus and axially) are depicted in different colours.

**Figure 6:**
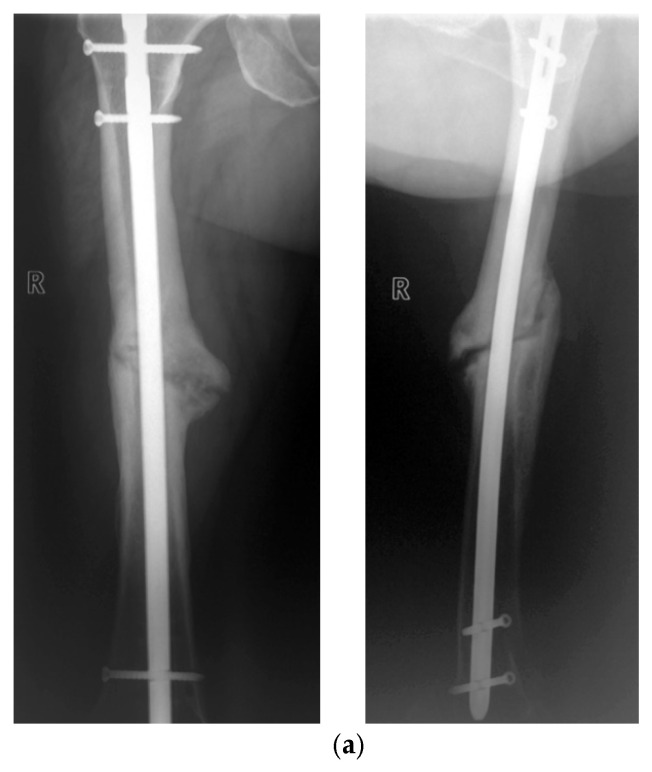

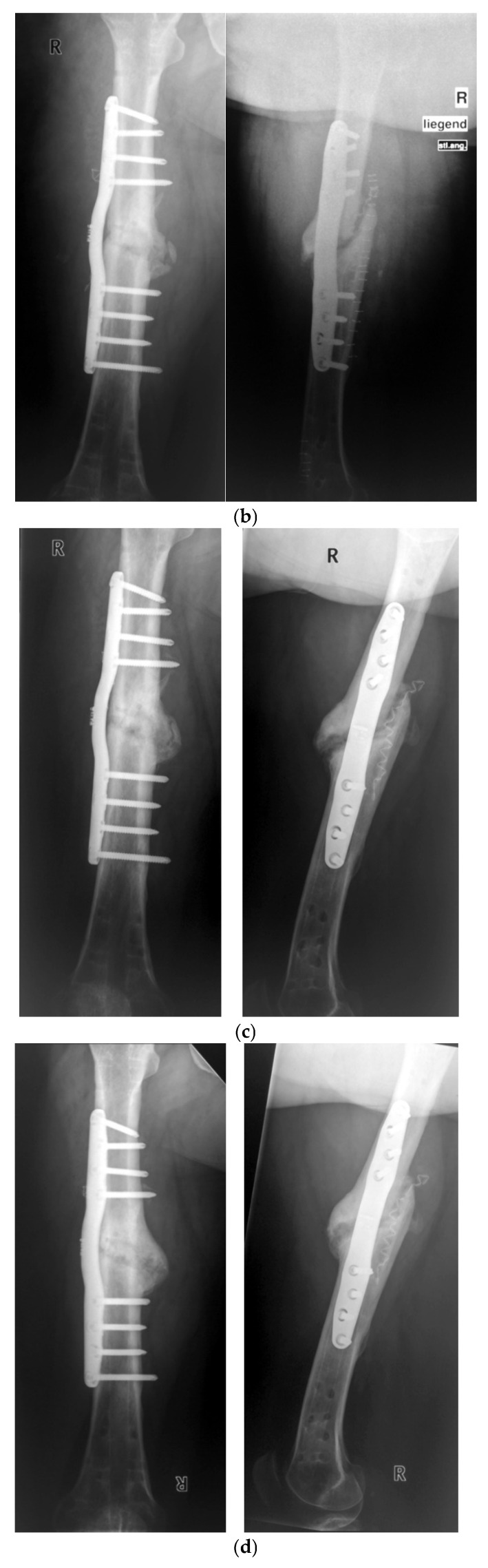

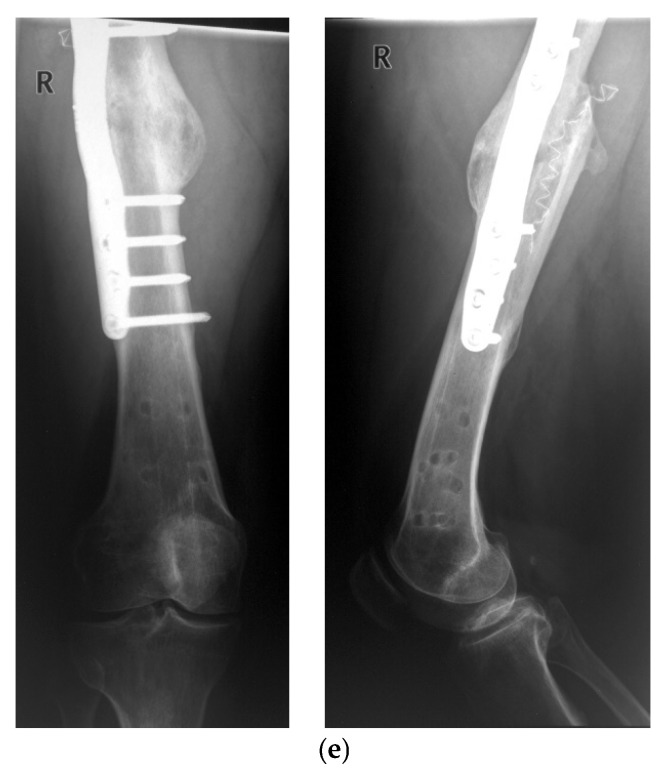
Anteroposterior radiographs preoperatively with a nail inserted (**a**), 5 days postoperatively (**b**), 8 weeks postoperatively (**c**) and 16/20 weeks postoperatively (**d**,**e**) are shown.

The example of a fast-healing non-union based on the measurement of elasticity with the smart implant is shown in Figure 5 and the corresponding radiographs in Figure 6. The 46-year-old patient was treated with a smart implant two years after the initial treatment due to a lack of bone healing, following a fracture of the nail from the initial treatment and a new treatment with an intramedullary nail with subsequent infection.

The healing process of one patient was extremely slow and took overall more than 10 years to full bony consolidation. The male patient, who was 46 years old at the time of the accident, was involved in a high speed road traffic accident and sustained intracerebral hemorrhage, hemorrhagic shock, cerebral edema, a dens fracture, fractures of three lumbar vertebrae without neurology, a lesion of the cervical roots C5–C7 right with mild motoric deficiencies, a blunt bilateral chest trauma, dislocated serial rib fractures, a splenic tear, a forearm fracture on the left, femur fractures on both sides, an open fracture of the lower leg and was initially on long-term ventilation. Two days after the accident, the patient underwent osteosynthesis of the bilateral femur fractures using an external fixator, closed reduction in the tibial shaft fractures and open reduction in the left radius and ulna fractures using plates. The above-mentioned fractures were surgically revised after 11 and 14 days, respectively. Both femoral fractures were treated by osteosynthesis plate.

Six weeks after the accident, the patient was initially transferred to the intensive care unit of our institution for further treatment. After 11 months post-accident, radiographs revealed the lack of bone healing. Therefore, the osteosynthesis plate was removed, non-union resection was performed and a re-osteosynthesis with a locked instrumented implant (smart implant) followed. During the surgery, autologous cancellous bone graft was used and an antibiotic chain was prophylactically inserted.

### Postoperative Procedure and Further Course

**Figure 7:**
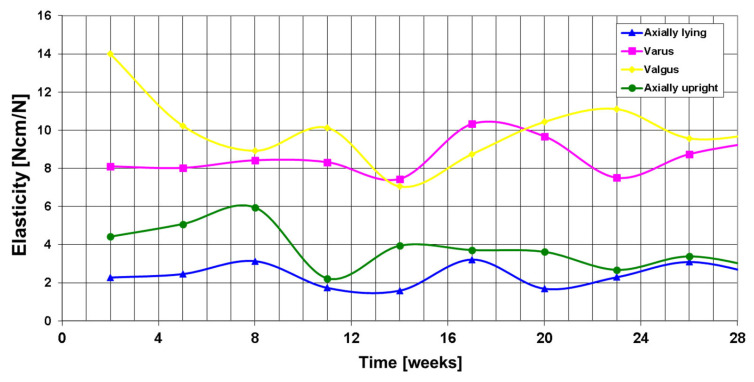
Elasticity measured under axial lying, varus, valgus and axial standing load during the first 28 weeks postop. No healing is detected.

**Figure 8:**
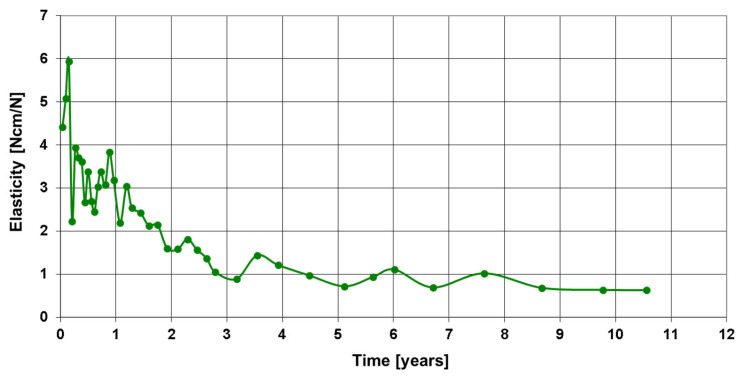
Elasticity of the osteosynthesis, measured under axial standing load, over the course of 10.5 years.

Figure 7 shows the telemetric measurements of all four load cases in the first 28 weeks after surgery and Figure 8 the entire course over 10.5 years.

**Figure 9:**
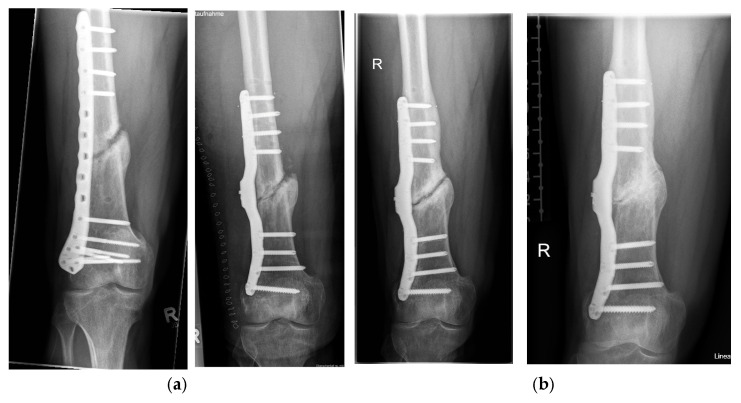

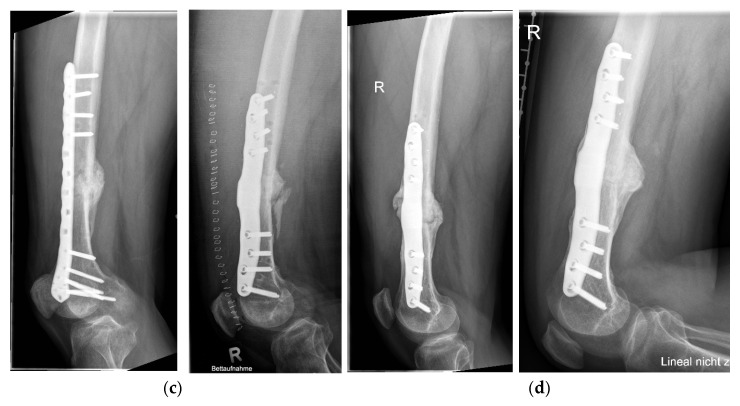
The figure shows the series of plain radiographs during very slow healing. Axial and anteroposterior radiographs showing the pre- (**a**) and postoperative situation (**b**), the situation at 3 years with few radiographic healing signs (**c**) and the late result after 10.5 years (**d**).

**Figure 10:**
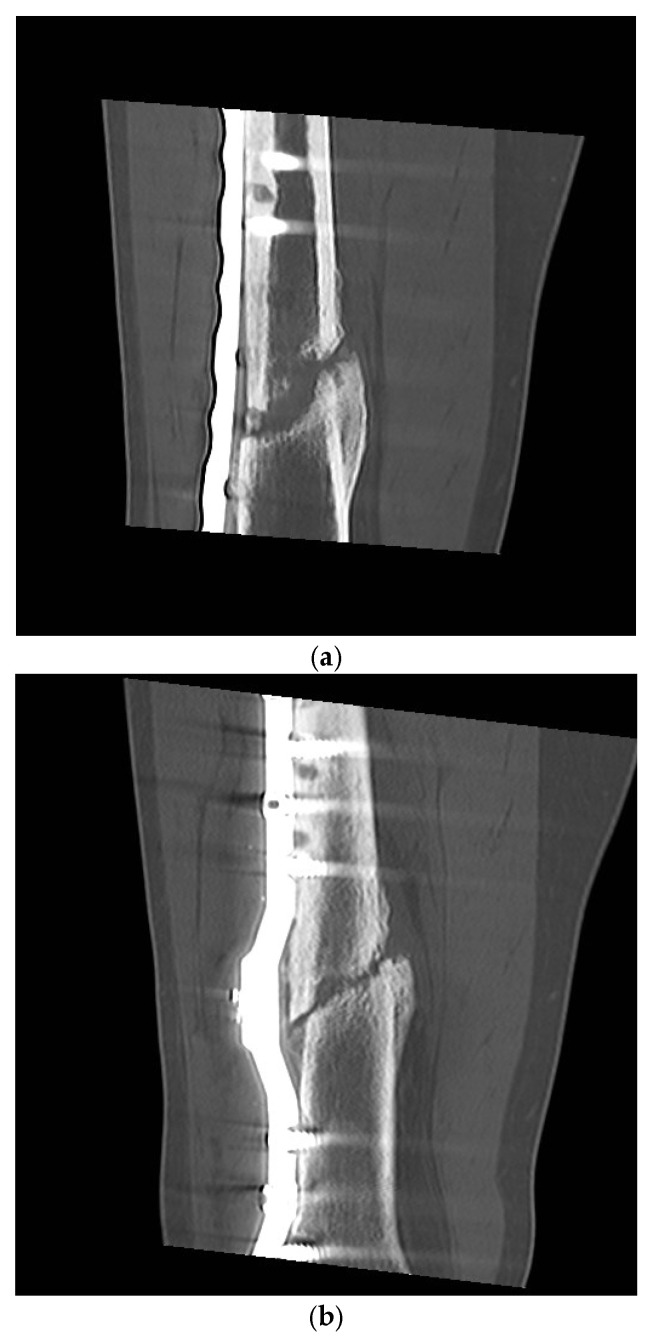

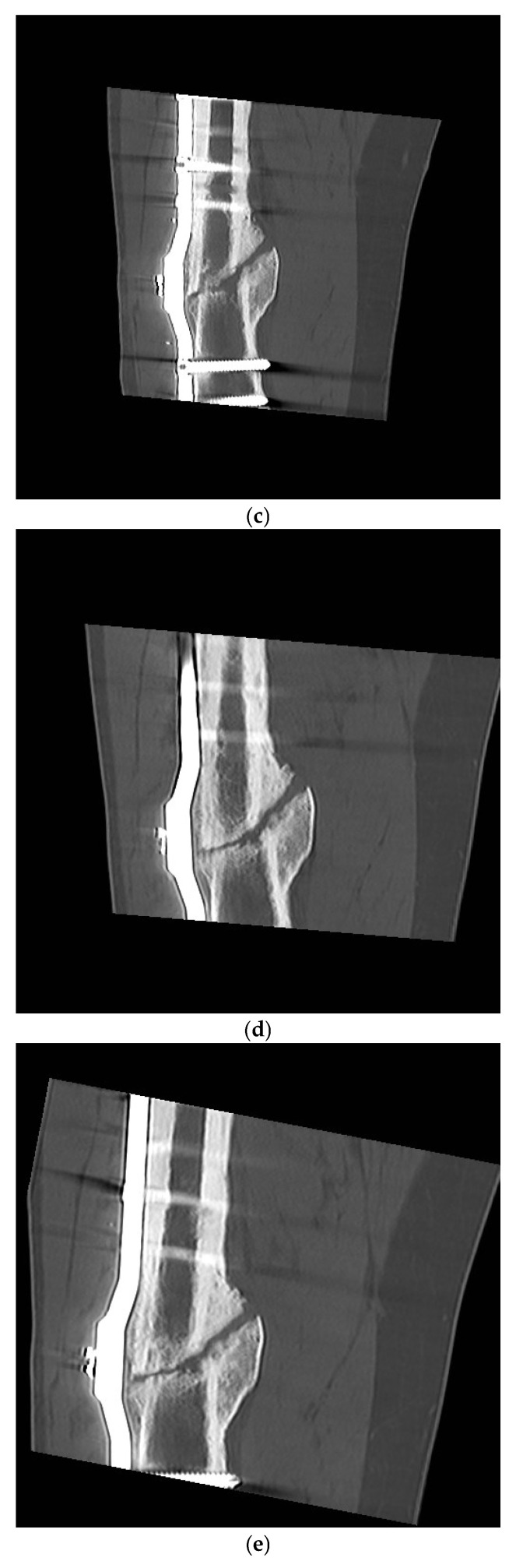

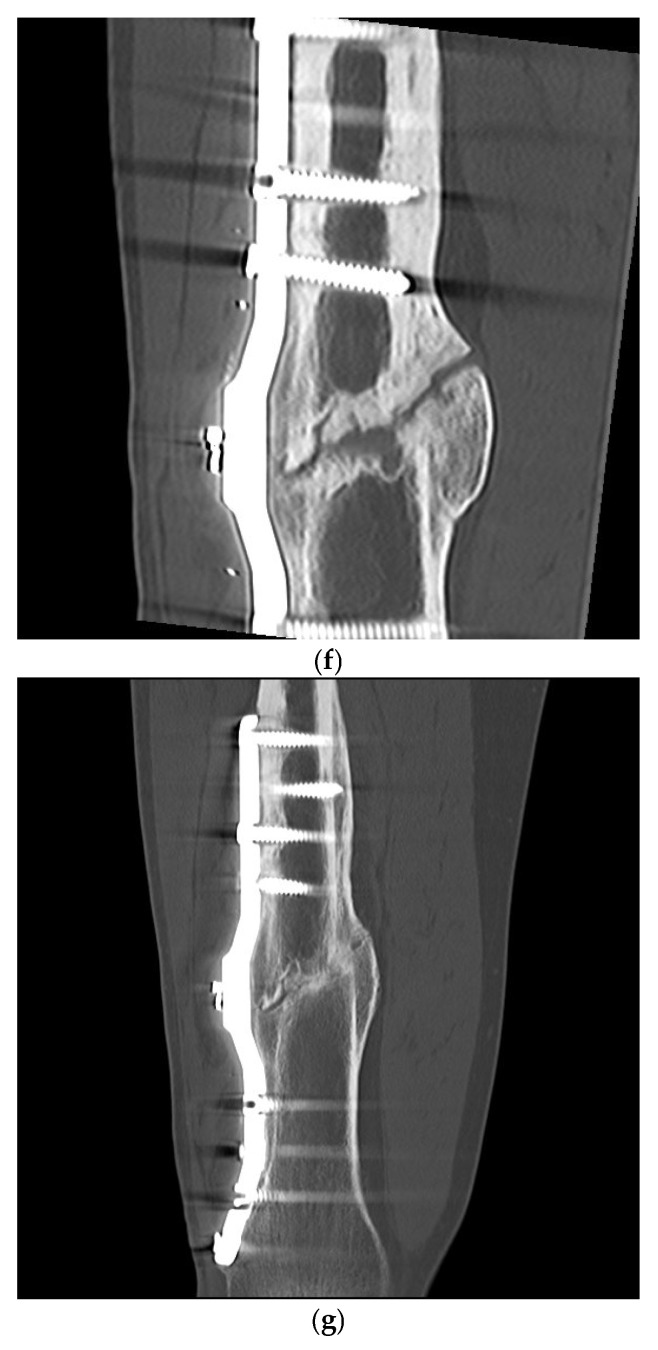
Computed tomography examinations over the course of time, preoperative (**a**), postoperative situation after 26 and 38 weeks (**b**,**c**), the situation after 1, 1.5 and 3.5 years (**d**–**f**), and the last result after 10.5 years (**g**). A coronal reconstruction is shown.

For 8 weeks after the operation with the smart implant, the patient was instructed to partially weight bear with a maximum partial load of 10 kg. The CT scan 13 weeks after surgery of the right femur showed only minimal, punctual deformities in the area of the non-union revision with the smart implant in place. The measurements of the elastic compliance of the osteosynthesis showed a slight improvement in the measurements (Figure 7), so that ultimately full weightbearing was advised. The radiographs and CT scans during the healing process are shown in Figure 9 and Figure 10.

After 26 weeks, the CT scan of the right thigh shows no further degeneration (elasticity axial standing 3.4 Ncm/N). Overall, there was still very little bony reaction in radiographs. However, the patient was largely symptom-free. After 32 weeks, although full weightbearing was possible over short distances, the gait pattern was by no means smooth (elasticity axial standing 2.6 Ncm/N). Radiologic examinations did not show any significant increase in the thickening.

After 38 weeks, the CT check of the right femur again showed no significant increase in union compared to the follow-up checks after 14 weeks and after 26 weeks. The measurements of the elastic compliance of the osteosynthesis also gave no indication that the stability in the area of the right femur had increased (elasticity axial upright 3.4 Ncm/N). It was discussed with the patient that a non-union revision in the area of the right femur with renewed cancellous bone transplant should be performed. The patient refused further surgical intervention; for this reason it was decided to continue with the monitoring process via the smart implant.

At 50 weeks, the load absorption values of the intelligent implant had decreased substantially. At that time, 40% of the axial load was still being absorbed via the plate, whereas 60% was already being absorbed via the bone. The varus and valgus stress tests were slightly worse with an average of 60% load transfer via the plate. Despite the lack of CT morphological evidence of a major tendency towards further deformity, the measurements showed a small decrease in load transfer via the plate such that further consolidation had to be assumed, which could not yet be detected morphologically on radiographs or CT scans.

Two years after the smart implant operation, the patient was reintegrated at work whereby his workplace had been altered to a desk position.

Three and a half years after surgery with the smart implant, there were no load-dependent complaints or clinical signs of instability in the area of the non-union. The measurements with the smart implant roughly corresponded to the previous findings; the measurements show a non-union that is not yet consolidated, but no increase in the sense of an expected complication due to instability. With the position and material position of the smart implant unchanged, there was no further increase in bony consolidation, the fracture edges were rounded and there were small cysts in the fragment ends. In summary, there was still what would be described as a hypertrophic non-union situation.

The telemetric measurements revealed a very slow but continuous decrease in the relative elastic compliance in the following years, such that a progression of healing could be assumed from the telemetrics.

The telemetric measurements in September 2024, 10.5 years after treatment with the smart implant, showed that all four load cases had decreased compared to the previous years, such that a radiological check was carried out both with native radiographs (Figure 9d) and computer tomography (Figure 10g). This revealed advanced endosteal and periosteal consolidation of the femoral non-union with only a partially rounded remnant of the former non-union gap still visible close to the lateral plate. The radiological findings thus confirmed the telemetric measurements of the smart implant but with a time lag of about two years.

The patient has been discharged from medical treatment but has agreed to come for a voluntary annual measurement to assess long-term function of such an implant.

## 4. Discussion

Based on these results, it can be concluded that monitoring bone healing using a smart implant is a good way of supporting the assessment of bone healing in difficult cases.

The conventional method of diagnosis is based on clinical and radiological criteria. Using radiographs, bone healing is defined by the presence of callus formation, cortical bridging and the disappearance of fracture lines [25]. In the case of non-union, healing is difficult to assess on the basis of native radiographs due to the irregular fracture gap running obliquely to the radiographic planes [22]. Therefore, computed tomography examinations are usually required to assess the healing process in non-unions [26]. In addition, several studies have shown that radiographs are not reproducibly assessed by clinicians [14,15].

Various methods have been developed to measure absolute fracture stiffness, mostly using sensors to measure deformation in the form of strain gauges, load cells, micrometers or protractors on external fixators [27,28,29,30,31,32,33]. However, only a few studies have been carried out on internal fixators [34,35,36].

What is new is that the healing process can be measured mechanically and quantitatively by telemetry using the instrumented internal fixator (Figure 1 and Figure 2). This is a system developed at the BG Klinikum Hamburg [18,19,20,21]. The bending load of the implant is measured when defined loads are applied to the extremity. This bending load decreases as the callus stiffness increases during the healing process. This system was used in patients with femoral non-union, so that quantitative mechanical data on bone healing is available in addition to the clinical/radiological findings.

More recently, the AO Foundation has developed a medical device to record the relative load of an implant that can be applied intraoperatively to a plate to treat fractures [37,38]. Animal experiments with the system have confirmed the possibility of quantifying the healing process.

Several recent review publications describe the development of so-called “smart implants”, defined as implants with measurement sensors, as an important future innovation opportunity [39,40].

This is the first time that the smart implant is still measuring correctly after 10 years and that it is therefore still possible to monitor the healing of a non-union after 10 years. The slow healing process calls into question the usual approach of revision surgery after a defined period of time. Rather, a more specific differentiation of the non-union and its healing progress seems necessary, which can be achieved with smart implants.

The case presented also shows that the measurements can be more sensitive than radiological procedures. The measurements are an ideal complement to conventional procedures when a new revision is required. Further advantages are the reduction in radiation exposure and the possibility of being able to assess the progression non-invasively at any time, as well as the direct measurement of the essential property of osteosynthesis and the indirect measurement of the essential function of the bone—the transfer of loads.

Fracture healing monitoring has seen significant advancements with the integration of artificial intelligence (AI) technologies. Recent studies indicate that AI can enhance the assessment of fracture healing by analysing radiographic images and patient data [41]. Machine learning algorithms are employed to identify patterns in imaging data, which may indicate the progression of healing more accurately than traditional methods.

AI models can predict healing outcomes by evaluating various factors such as age, comorbidities, and imaging features. For instance, convolutional neural networks (CNNs) have been effectively used to classify the stages of fracture healing in X-ray images, providing clinicians with valuable insights into the healing process [42]. Additionally, AI-driven tools can help in early identification of complications, thereby facilitating timely interventions.

Moreover, wearable technology and mobile applications leveraging AI algorithms are increasingly used to monitor patient-reported outcomes and activity levels, correlating these with clinical healing parameters [43,44]. As the technology of smart implants as well as AI progresses, it can be anticipated that this will revolutionize the field of orthopedic healing monitoring, leading to improved patient outcomes and personalized treatment plans.

## 5. Conclusions

### 5.1. Major Findings

Smart implants that continuously monitor mechanical load/strain at a fracture site provide objective, patient-specific information that supports clinical assessment in difficult or ambiguous cases. This technology bridges the gap between intermittent radiographic assessment and real-time functional data.

Continuous fracture monitoring can substantially reduce the need for repeated radiographs: objective load/strain trends allow clinicians to confirm progressive healing or detect stagnation without routine X-rays, lowering radiation exposure and follow-up costs. Relevant recent work on sensor-enabled implants and reconstruction plates demonstrates feasibility for wireless load/force measurement.

Non-union is not strictly time-dependent: fractures classified as non-unions by fixed time windows can consolidate even after prolonged periods without radiographic progress. Time-based definitions of non-union are therefore limited and should be re-examined. Continuous mechanical load curves offer a more direct, biologically relevant metric of healing potential and mechanical competence.

### 5.2. Clinical and Health-System Implications

Reoperations for presumed non-union can be reduced: earlier and more accurate detection of true mechanical failure vs. slow but ongoing consolidation allows better timing of interventions and avoids unnecessary surgery.

Rehabilitation can be individualized and potentially shortened in uncomplicated cases: clinicians can advance weightbearing and therapy based on measured load transfer rather than conservative fixed protocols.

Resource use and costs may decrease through fewer radiographs, optimized clinic visits, and fewer avoidable reoperations; patients benefit from less radiation and more tailored recovery plans.

### 5.3. Technological Vision and Platform Development

An open, device-agnostic smart-implant platform (suitable for plates, nails and joint replacement devices) that streams load/strain data to a centralized platform is feasible and desirable. Key capabilities should include secure wireless data transfer, standardized data formats (so different implant types can be compared), clinician dashboards and patient-facing summaries. Examples in the literature demonstrate plate/nail and active implant concepts that measure forces and provide actionable metrics.

## Data Availability

The datasets generated and analyzed during the current study are not publicly available due GDPR restrictions but are available from the corresponding author on reasonable request. All data generated or analyzed during this study are included in this published article.
